# The Longevity of the Gonococcus1Read at the thirty-third annual meeting of the Medical Association of Central New York, held at Rochester, N. Y., October 16, 1900.

**Published:** 1900-12

**Authors:** E. Wood Ruggles

**Affiliations:** Rochester, N. Y., Member of the Rochester Academy of Medicine, the Rochester Pathological Society and the Monroe County Medical Society


### THE LONGEVITY OF THE GONOCOCCUS.[1]

By E. WOOD RUGGLES, M. D., Rochester, N. Y.,
Member of the Rochester Academy of Medicine, the Rochester Pathological Society and the Monroe County Medical Society.

THE title of this paper of itself precludes any very ambitious effort and its only purpose is to increase, to some extent, the mass of evidence which is constantly accumulating, concerning the virulence and the tenacity of life of the gonorrheic virus. It must be admitted by all that, while these facts are perhaps well enough known, theoretically, by the general practitioner, they are far from being thoroughly realised and acted upon.

Recent German authorities compute that gonorrhea is, next to measles, the most prevalent contagious disease, about three quarters of the adult male population and, according to different observers, from one-sixth to one half of adult females having contracted it. The proportion is probably higher in Germany than here, since coitus is there regarded as the natural means of voiding a secretion—a purely physiological function, like defecation and urination. They are ignorant of or disbelievers in there being any morality or immorality

1. Read at the thirty-third annual meeting of the Medical Association of Central New York, held at Rochester, N. Y., October 16, 1900.

about the act, before marriage, so far as the male sex is concerned. This attitude, of course, tends to the increase or rather universality of prostitution, aided by the fancied, but illusory protection afforded by the regular medical examination of prostitutes.

The provident German father seeks only to avoid incoherence and excess in the exercise of the function, by warning his son against too early and too frequent indulgence and tries to avoid the danger of infection by securing for him a good-principled and constant mistress.

On the other hand, the American and English standpoint, that fornication is immoral, shames very many into concealing their venereal ailments, and so the proportion of such sufferers is much underestimated.

As regards the virulence of the gonococcus and the variety of lesions caused by it, new facts are being constantly demonstrated. Deaths ascribed thereto are more and more frequent, and staining and culture experiments have confirmed its presence in suppurating buboes, in the epididymis, in palmar abscess, in the ovaries, tubes and peritoneal cavity, the pleura, the endocardium and the arterial blood, the brain, meninges and spinal cord, the muscles, joints and tendon-sheaths, and the like.

Strangely enough, though the diagnosis of gonorrheal nephritis is frequently made, there have been only three authenticated cases of the gonococcus being found in the kidney. There is no doubt about its causing nephritis, but it is generally through the medium of other bacteria. The bladder also, like the adult vagina, is, as a rule, immune to the gonococcus, which does not thrive on squamous epithelium. In regard to the longevity of the gonococcus authorities differ. White and Martin's text-book on genito-urinary diseases, one of the best works yet published, says:

> Young married women become infected because long standing gleet is not generally regarded as a possible bar to matrimony; hence men with chronic urethral discharge should at least understand that the gonococcus may persist and maintain its virulence for two or three years.

I have been able to find no authority which mentions the persistence of the gonococcus, in the probable absence of reinfection, for more than four or five years, and many believe that these cases are caused by a disregarded or unacknowledged new infection. Finger mentions finding the gonococcus in two cases, four and four and a half years after infection, but they had both practised coitus repeatedly during the time.

The case I am about to relate covers twice this length of time and may be regarded as open to the same doubts, but the patient is an intimate acquaintance of mine and I feel certain would not lie to me, beside having no motive in so doing:

X. Y., 34 years old, heredity and general health good. Married nine years and has one healthy child. First gonorrhea in 1883; lasted two or three months; second gonorrhea, fall of 1885. Had anterior and posterior urethritis, epididymitis and cystitis, all of extreme severity. The strangury was so excessive that he was obliged to "camp out" on a water closet for forty-eight hours and empty his bladder drop by drop. The acute symptoms lasted ten or twelve weeks. Then had gleet for two years with exacerbations following drinking, railway journeys, etc. In 1887 had apparently another acute attack following coitus. Since 1887 has been free from discharge, but clap shreds have always been present in urine.

Married in 1891. Had not practised intercourse for over a year before marriage and has had no extra-marital relations, so that the last exposure dates from at least ten or eleven years ago. His wife has never been infected; has had leucorrhea but once, and that during the presence of an enormous abdominal tumor, which would naturally press upon and congest all the pelvic organs. When this tumor was removed, both the ovaries and the tubes were carefully examined and found to be perfectly healthy. She has never had pain on urination, bladder, tubal or ovarian symptoms.

The evening of August 20, 1900, he drank several cocktails and three or four glasses of beer. The same night practised coitus and, owing to the alcohol, it was extremely protracted. Here then is the explanation of the auto-reinfection and subsequent symptoms, the stimulus of alcohol, especially in the form of beer upon the genito-urinary tract, followed by the unaccustomed duration of the congestion and irritation of the parts. Small wonder that the latent prostatic gonorrhea reasserted itself, and that the next morning there was some urethral irritation and a very slight discharge. The succeeding morning, August 22d, he came to my office. The meatus was somewhat reddened, and there was a very slight muco-purulent discharge. On staining found very many pus and a few epithelial cells, many pus cells containing unmistakable gonococci. Urine, first portion, clear, with clap shreds; second portion, clear. Prescribed injection of ½ per cent. protargol solution.

August 26th, he said the discharge had soon stopped, but by milking the urethra, I obtained a muco-purulent discharge, containing pus and gonococci. On rectal examination, I found prostate to be over two inches in diameter, round, not very tender, and exactly resembling the hypertrophied prostate of old age, even in firmness of texture, though under massage it became softer; no fluctuation. The secretion expressed contained much pus, a few of the pus cells enclosing gonococci. Patient's condition was unchanged till September 1st (there was at no time pain in the urethra, even on urination, nor in the prostate) when he developed headache, general malaise and fever. I first saw him September 3d, owing to his absence from town, when I found him with flushed face, full pulse and temperature $102\frac{3}{5}°$. There was a muco-purulent discharge, containing pus and gonococci. He then urinated, urine being very cloudy, and the effort of expelling the last drops brought a new gush of pus, with gonococci. This evidently came from the prostate, the urethra having been just washed out by the passage of urine. The fever ran intermittently till September 7th, when I again milked the prostate. The next day he resumed work. On September 13th massage of prostate, anterior injection of 1 per cent. protargol solution and instillation behind sphincter of $\frac{3}{4}$ per cent. solution. After this he complained of pain and irritation.

September 15th he developed left epididymitis, evidently subacute, as the pain was trifling and fever low, 101°. First and second glasses of urine cloudy. September 17th, epididymis little painful, except on pressure; forms a round mass, two-thirds the size of testicle and freely movable upon it, as if connected by a pedicle. Temperature, $102\frac{1}{5}°$; abundant urethral discharge, somewhat bloody. It is almost unnecessary to mention that in acute gonorrheal epididymitis, the urethral discharge suddenly stops, to recommence when the attack is over. September 19 and 20th, no pain or fever; epididymis very little tender to pressure. Muco-purulent discharge containing pus and gonococci. Prostatic secretion shows a lot of pus and epithelial cells and a few diplococci. September 28th, slight discharge, containing pus, epithelium and mucus; no bacteria. First glass of urine clear with filaments; second glass, clear; prostate considerably smaller, secretion less purulent, no bacteria.

October 2d, considerable inspissated discharge at meatus, looking as if squeezed up from prostate. This patient has been able to expel material from the prostate by voluntary muscular effort, during the whole attack. Also at the end of urination and defecation prostatic secretion very often appears at the meatus. First portion of urine cloudy with filaments; second, slightly cloudy. Prostate rather larger and distinctly boggy in one portion, from which I expressed considerable secretion, which was much less purulent; no bacteria. October 10th, both urethral and prostatic secretions still contain a few gonococci.

This patient is now doing his regular work and feeling well, but it will take some time to clear up the long standing prostatic inflammation.

November 12th. There is no longer any discharge, the urine is clear, though with filaments, and the prostate greatly reduced in size. No bacteria have been found in its secretion or in the urinary filaments since October 2d.

It may be claimed that the massage of the prostate caused both the first febrile attack and the subsequent epididymitis. This is quite possible, but at present no other efficacious means of treating chronic prostatic inflammation is known, and it was certainly better to risk local infection by the expressed secretion than to leave this constant focus of poison intact.

This case is of particular interest on account of the persistence of the gonococcus for more than ten years, in the absence of all suspicious coitus. The finding of gonococci in a case of gleet, ten years after the primary gonorrhea, in a man repeatedly exposed to reinfection, proves absolutely nothing as to their being lineal descendants from the first inhabitants. It is far more likely that there have been several fresh colonisations by the germs. But since such chronicity, with absence of all noticeable symptoms, is possible, and since the ravages of this disease, commonly regarded as so insignificant, are so widespread and terrible, affecting not only the guilty but the innocent, it certainly seems high time that its gravity should be recognised, its diagnosis accurately made, its treatment carefully performed and its cure scientifically assured by the use of several tests. It is impossible to fulfil these indications without the constant use of the microscope, and I will close by quoting from a former paper of mine, on a similar subject:

There is not the slightest doubt that it would be exactly as just to our patients to treat any febrile disease without the clinical thermometer, as to treat gonorrhea without the frequent, almost daily use of the microscope.

294 Alexander Street.

